# Supplementary material for: Evolutionary Adaptation by Repetitive Long-Term Cultivation with Gradual Increase in Temperature for Acquiring Multi-Stress Tolerance and High Ethanol Productivity in Kluyveromyces marxianus DMKU 3-1042
Source: Microorganisms. 2022 Apr 9;10(4):798. doi: 10.3390/microorganisms10040798 (PMC9032449; doi:10.3390/microorganisms10040798)
Supplement: Supplementary file 1 [file microorganisms-10-00798-s001.zip › microorganisms-1654758-supplementary.pdf]

microorganisms

Supplementary Materials

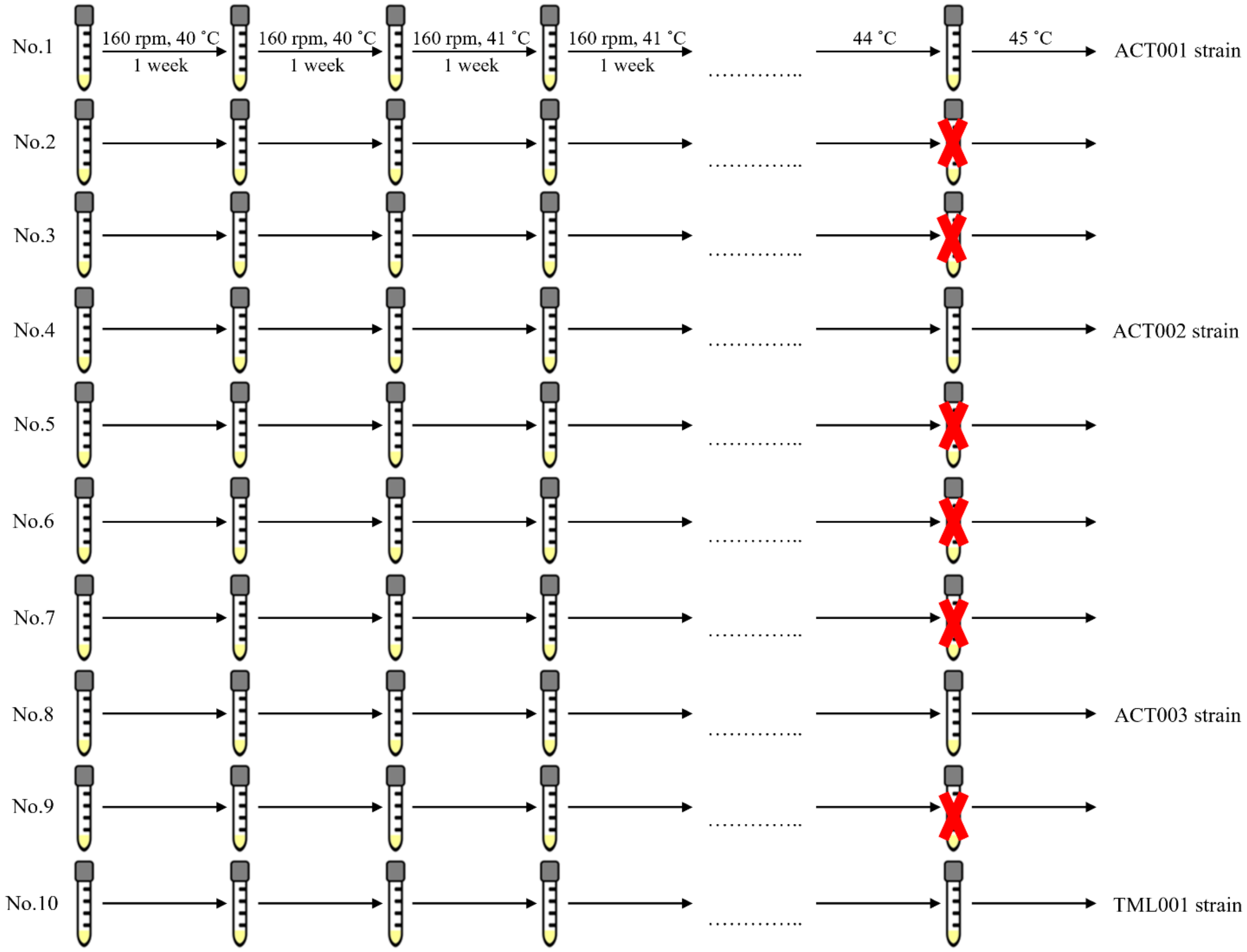

Figure S1. Schematic diagram of RLCGT for *K. marxianus* DMKU 3-1042. *K. marxianus* DMKU 3-1042 was subjected to RLCGT in YPD medium at temperatures from 40 ˚C to 45 ˚C, and finally 4 adapted strains were obtained. Cultivation for 7 days at each temperature was performed twice. Details are given in Materials and methods.

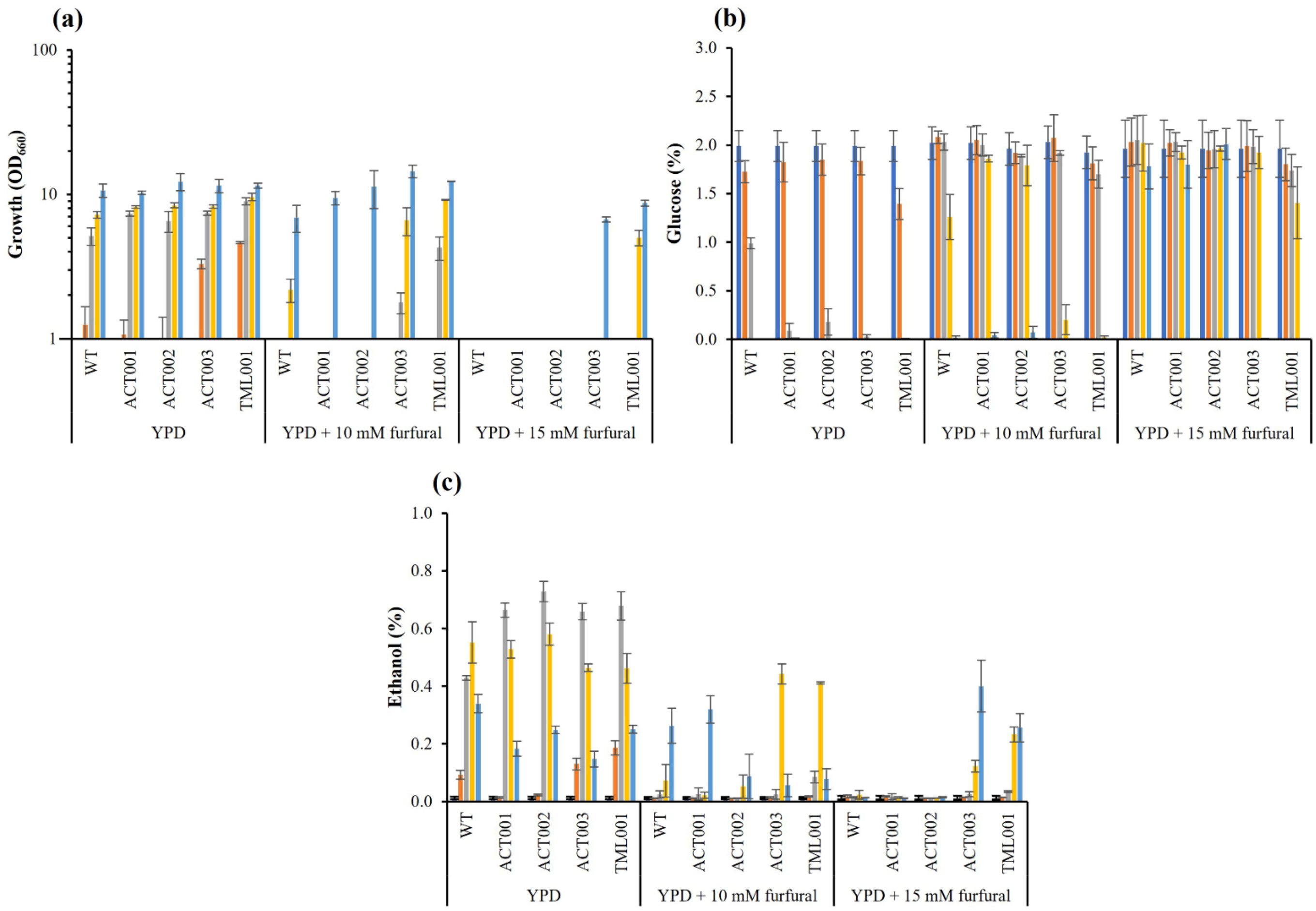

Figure S2. Effects of furfural on growth and fermentation parameters of adapted strains at 45 ˚C. Adapted strains were cultivated in YPD medium supplemented with 10 mM or 15 mM furfural at 45 ˚C under a shaking condition at 160 rpm for 48 h. Growth **(a)** was determined by measuring OD_{660}. Glucose **(b)** and ethanol **(c)** were determined by HPLC. Error bars represent ± SD of values from experiments performed in triplicate.

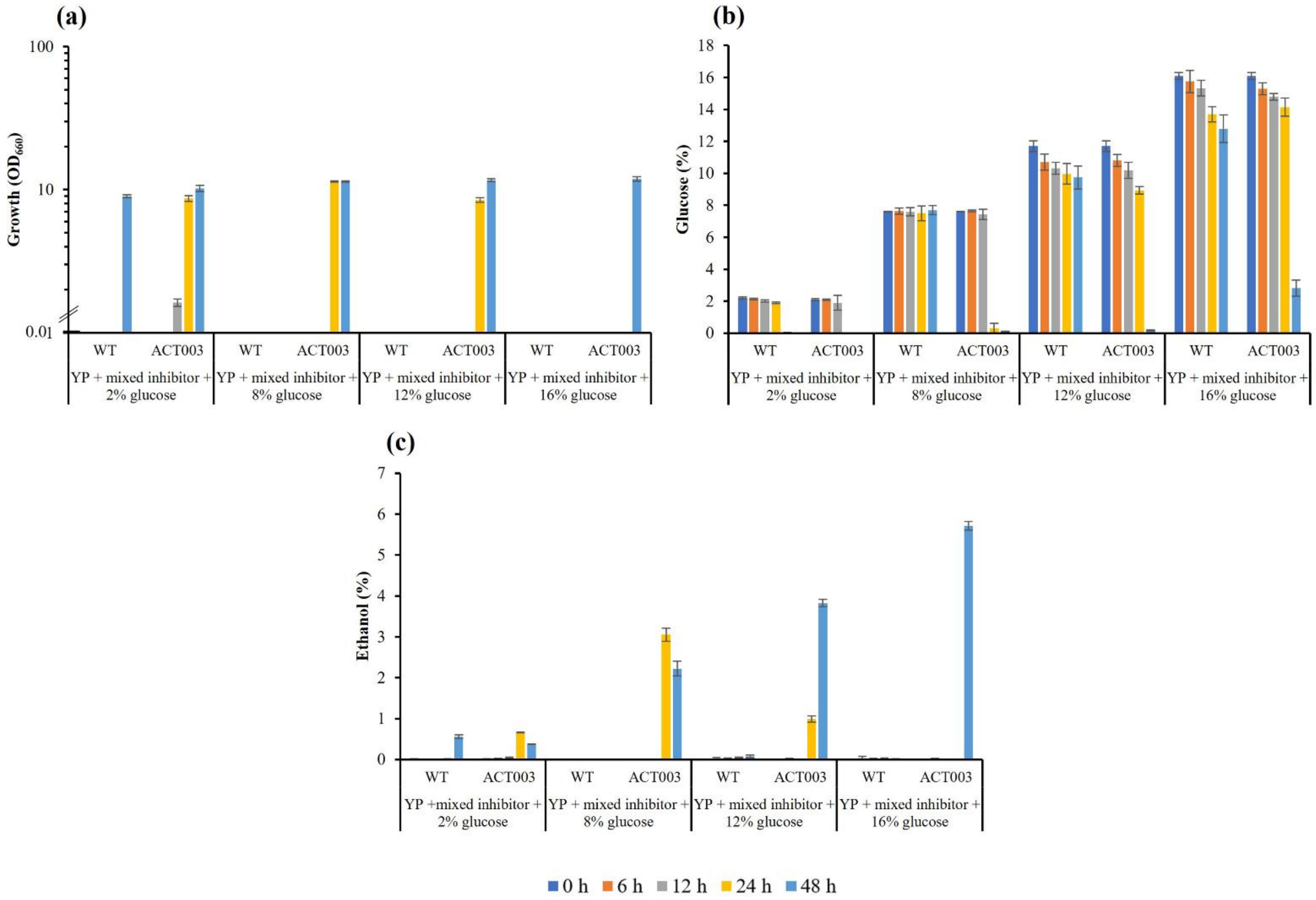

Figure S3. Effects of multiple inhibitors on growth and fermentation parameters of adapted strains at 45 °C. Adapted strains were cultivated in YPD medium supplemented with three inhibitors of 0.15% acetate, 7.5 mM furfural and 0.075% vanillin and with 8%, 12% or 16% glucose at 40 °C under a shaking condition at 160 rpm for 48 h. Growth **(a)** was determined by measuring OD_{660}. Glucose **(b)** and ethanol **(c)** were determined by HPLC. Error bars represent ± SD of values from experiments performed in triplicate.

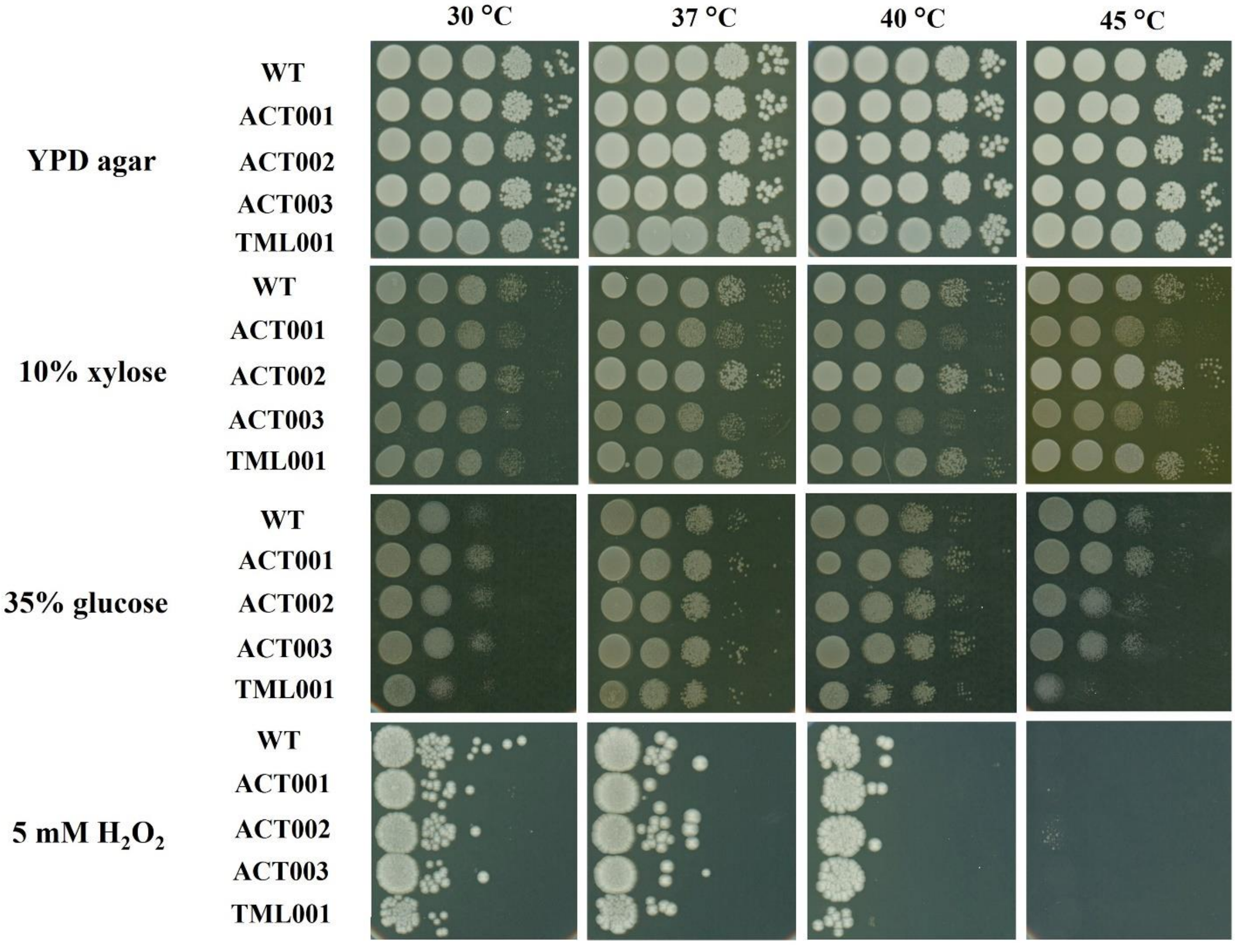

Figure S4. Characterization of adapted strains. Growth of adapted strains on YP agar plates supplemented with 10% xylose and YPD agar plates supplemented with 35% glucose or 5 mM H_2O_2 were compared. The plates were incubated at 30 °C - 45 °C for 48 h.

Table S1. Primers used in this study.

No.	Primer name	Nucleotide sequence
1	ACT1-1552906-ins-5'-F	ATCACTCTTTCGGTGTTGCTC
2	ACT1-1552906-ins-3'-R	GTCATAGACAACGTACATCCTCTG
3	ACT1-47658-del-5'-F	TTAAGGGCATTAGGTGGCCC
4	ACT1-47658-del-3'-R	GGGAAGGTGGAATTAGTAACTTG
5	ACT2-1161817-snv-5'-F	GAAAGCAGTACTTCGTTTGGAC
6	ACT2-1161817-snv-3'-R	GGTTGGGATCGTATTGACGAAT
7	ACT2-712698-snv-5'-F	CGGTTATACTCTGGACCAAGC
8	ACT2-712698-snv-3'-R	CATTCTTGACTGACTCCACGGTG
9	ACT2-385614-snv-5'-F	GCTTCTTCGGAGGAATACCTTC
10	ACT2-385614-snv-3'-R	ACCAAGAGAACTAGAGTGTTCGG
11	ACT2-1269738-snv-5'-F	ACAATTGTGCACCCAAGAGCT
12	ACT2-1269738-snv-3'-R	GACGGCACTGGGAGCGTA
13	ACT3-1107715-snv-5'-F	GTCTCCCGTTACCAACAAGTC
14	ACT3-1107715-snv-3'-R	TCTCCCTTCCTTTCAAACCTG
15	TML1-613476-snv-5'-F	TACGAGGCTTGCGATCCCT
16	TML1-613476-snv-3'-R	GATGCACCGAATGATTTCTTGG
17	TML1-968552-ins-5'-F	CGAAGTTGTCCCAGGTGATATC
18	TML1-968552-ins-3'-R	CAGCACCGACAGCCATGG
19	TML1-1010140-snv-5'-F	GGCATCTACGTGAACGCTTC
20	TML1-1010140-snv-3'-R	CTTCATACCAACCACACAGGG
21	TML1-1714543-snv-5'-F	GTACTTCCGCCTGCAGTGT
22	TML1-1714543-snv-3'-R	CCACGCCGGAATTGTCGC
23	TML1-767036-snv-5'-F	CATGAGGAGACAGCAGTAGCT
24	TML1-767036-snv-3'-R	TTGAAGGCGCCACCGCCA
25	TML1-724738-snv-5'-F	GTGCCTCTGGATCGTAATACG
26	TML1-724738-snv-3'-R	TCGGGCTCGTTGGTTCCAATTA
27	TML1-815396-snv-5'-F	ACGGCGTTGGTCTGCCTC
28	TML1-815396-snv-3'-R	GGTGGCCTTTGCACAACGG

Table S2. Comparison of evolutionary adaptation in this study with evolutionary adaptations reported previously.

Stress	Condition	Parental strain	Adapted strain	Increased ethanol (%)	Phenotype	Ref
Temperature (In addition, ethanol, acetic acid, other by-products and nutrient starvation)	Repetitive long-term cultivation in YPD medium with a gradual increase of temperature from 40 ˚C to 45 ˚C	*K. marxianus* DMKU 3-1042	Four mutants (ACT001, ACT002, ACT003 and TML001)	20 – 40	- acetate tolerance<br>- formate tolerance<br>- acid tolerance<br>- ethanol tolerance<br>- furfural tolerance<br>- HMF tolerance<br>- vanillin tolerance	This study
Inhibitors in acid prehydrolysate of hardwood	Cultivation in acid prehydrolysate of hardwood supplemented with nutrients and minerals at 30˚C for more than 6 weeks. The cultivation was performed with a gradual increase of amount of the acid prehydrolysate.	*P. stipitis* NRRL Y-7124	One mutant	48	- acid tolerance	[26]
High sugar concentration (22 brix)	Sequential batch fermentation in sugar juice medium, 200 generations at 28 ˚C	*S. cerevisiae* G85 Chinese rice wine yeast	One mutant (G85X-8)	3	- ethanol tolerance<br>- osmotic tolerance<br>- temperature tolerance	[22]
Ethanol	266 nm laser radiation and repetitive cultivation in YPD supplemented with 11%-15% ethanol at 34 ˚C	*S. cerevisiae* YE0	One mutant (SM4)	29	- ethanol tolerance	[28]
Acetic acid	Cultivation in YPD supplemented with 0.3%-1.3% acetic acid at 28 ˚C for 33 days. (The cultivation period was short (1-2 days) up to 19 days and increased (3-5 days) after that).	*S. cerevisiae* Y8 industrial osmotolerant strain	One mutant (Y8A)	26	- osmotic tolerance<br>- temperature tolerance<br>- saline tolerance<br>- ethanol tolerance<br>- organic acid tolerance	[29]
Lactose concentration (200 g/L)	Cultivation in whey permeate (150-200 g/L lactose) at 37 °C for 65 days	*K. marxianus* MTCC 1389	One mutant	18	- osmotic tolerance	[4]
(Xylose as a carbon source)	Repetitive cultivation in YEP medium supplemented with 2% xylose at 45 ˚C for 24 h, 60 cycles	*K. marxianus* NIRE-K3	One mutant (K3.1)	16	- improved xylose utilization	[45]
(Xylose as carbon source and 2-deoxyglucose)	Repetitive cultivation in YP supplemented with 1.5% 2-deoxyglucose and 4% xylose at 30˚C, 6 times over 50 days	*K. marxianus* 17694-DH1	One mutant (SBK1)	NR	- improved co-fermentation of glucose and xylose	[48]
Ethanol	Ten consecutive rounds of liquid nitrogen freeze–thaw treatment followed by plate screening under osmotic and ethanol stresses	*S. cerevisiae* Y-1	One mutant (YF10-5)	16	- osmotic tolerance<br>- ethanol tolerance	[14]

Note: NR, no reported.
